# Giant sacral schwannoma: A report of six cases

**DOI:** 10.3109/03009730903359674

**Published:** 2010-04-07

**Authors:** Chanplakorn Pongsthorn, Hiroshi Ozawa, Toshimi Aizawa, Takashi Kusakabe, Takeshi Nakamura, Eiji Itoi

**Affiliations:** ^1^Department of Orthopaedic Surgery, Tohoku University Graduate School of Medicine, SendaiJapan; ^2^Department of Orthopedics, Mahidol University Faculty of Medicine Ramathibodi Hospital, BangkokThailand

**Keywords:** Pelvis, sacrum, schwannoma, subtotal excision

## Abstract

Sacral and presacral schwannomas are often found incidentally, because they present with vague symptoms or symptomless. Schwannoma occurring in this area occasionally presents with enormous dimensions, known as a giant schwannoma. The tumor removal is a surgical challenge due to the difficult approach and abundant vascularity. The aim of this study is to review cases of giant sacral schwannomas focusing the surgical management and outcome. Six patients with sacral and presacral schwannoma were treated surgically. The patients included two males and four females, and the mean age was 47.8 years. All patients experienced pain at the time of presentation. The tumors were classified as intraosseous type in one case, dumb-bell type in four cases, and retroperitoneal type in one case. The tumors were removed with a piecemeal subtotal excision in three patients, a partial excision in two patients, and enucleation in one patient. The surgeries were performed by the combination of an anterior and posterior approach in three patients, a posterior approach in two patients, and an anterior approach in one patient. The mean surgical time was 7.8 hrs, and the mean blood loss was 2572 g. The tumor recurred in one patient after the partial excision and was removed completely in a second surgery. No patient, including the patient who underwent the second surgery, presented with pain and obvious neurological deficit at the final follow-up. The surgical treatment of the giant sacral schwannoma with a piecemeal subtotal excision can achieve a good outcome, avoiding unnecessary neurological deficit.

## Introduction

Sacral and presacral tumors are uncommon and occur in approximately 1 of 40,000 hospital admissions (1,2). Schwannoma is one of the tumors that often occur in the sacral and presacral regions (3). It usually grows slowly. It is often found incidentally, because it presents with vague and non-specific symptoms (4,5). Most schwannomas are benign. Malignant schwannoma is very uncommon (6), and malignant transformation is exceedingly rare (7). Schwannoma occurring in this area occasionally presents with enormous dimensions, known as a giant schwannoma, and is difficult to manage.

Three types of giant sacral schwannomas have so far been described based on the anatomical findings: retroperitoneal schwannoma (8–12), intrasacral (osseous) schwannoma (13,14), and spinal schwannoma (dumb-bell tumor) (2,15,16). They show different clinical features. The retroperitoneal schwannoma usually presents with slow growth and does not show specific symptoms (5). Diagnosis and treatment are often delayed until the tumor enlarges. The intrasacral schwannoma is usually an intraosseous lesion. This tumor induces mild local pain. The pain occurs late in the development of the lesion. A neurological deficit is an unusual finding (13). In a few cases, it induces low back pain (14). The spinal schwannoma arises from spinal nerve root and presents as a dumb-bell tumor. Sacral dumb-bell schwannomas account for 4% of all spinal dumb-bell schwannomas (17).

This report presents cases of giant sacral schwannomas. The tumor characteristics and clinical pictures are reviewed. The problems associated with the surgical removal of this tumor are also discussed.

## Patients

Six patients with sacral and presacral schwannoma were treated surgically. The patients included two males and four females, and the mean age was 47.8 (range 38–58) years. The tumors arose from L5 or sacral roots in all patients. Plain X-ray, computed tomography (CT) scan, and magnetic resonance images (MRI) were examined before surgery. Angiography was performed in four patients.

### Case illustrations

**Figure 1.**
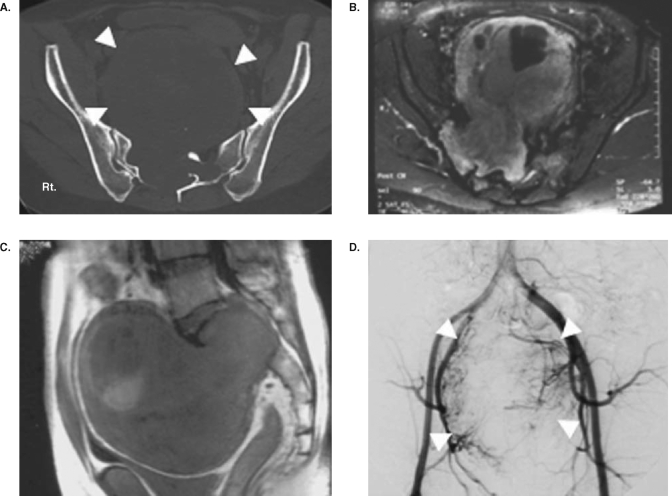
Case 2. CT (A) showing bony destruction of the sacrum and tumor outline (white arrow-head). Axial image (B) and sagittal image (C) of MRI showing the enormous tumor with heterogeneous signal intensity. Preoperative angiogram (D) showing a high vascularity of the tumor (white arrow-head) from both internal iliac arteries. The tumor was excised completely by a piecemeal excision with right S1 root sacrifice via the posterior and anterior approach.

*Case 2.* A 38-year-old male experienced right buttock pain for 23 years. Bilateral leg hypalgesia was revealed during a physical examination, but no motor weakness was observed. Plain X-ray and CT showed extensive bony destruction (Figure 1). MRI showed the presence of a tumor arising from the right S1 root and which extended through the sacrum body into the retroperitoneal space. The tumor showed heterogeneous iso/high intensity on T1/T2-weighted images (WI) with positive gadolinium­diethylenetriamine penta-acetic acid (Gd-DTPA) enhancement. A preoperative angiogram demonstrated feeding vessels from both internal iliac arteries. Surgery was performed by the combination of an anterior and posterior approach. The tumor was removed subtotally by piecemeal excision with sacrifice of the right S1 root. Motor weakness was found on the hamstring and ankle plantar flexor, and erectile dysfunction was also detected immediately after surgery. The neurological deficits were resolved completely 2 years after surgery, and the patient remained disease-free during the follow-up period.

**Figure 2.**
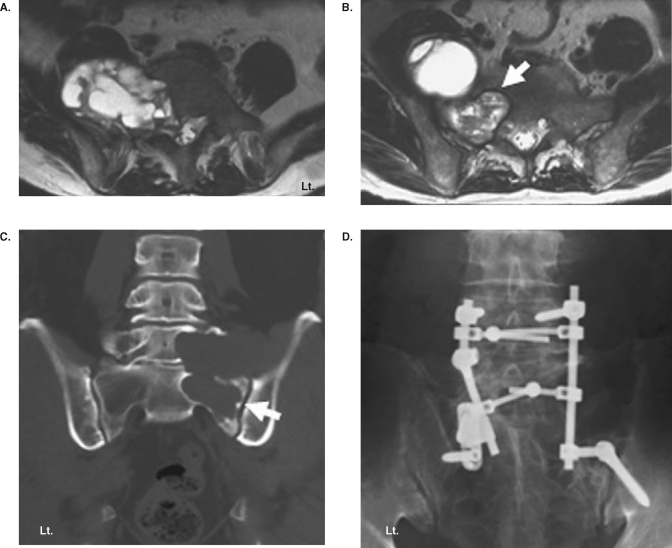
Case 5. Axial images of T2-weighted MRI at L5 level (A) and S1 level (B) showing bony destruction of the vertebral body and a tumor involving the intraosseous area of S1 (white arrow) and presacral region. Frontal reconstruction of CT (C) showing obvious bony destruction of the L5 body, sacrum, and sacroiliac joint (white arrow). Partial tumor excision was done using the posterior approach, and lumbopelvic fixation and bone grafting were performed (D).

*Case 5.* A 58-year-old male experienced buttock pain for 7 years. MRI and CT showed that the tumor with iso/high intensity on T1/T2 WI extending from the right L5 to the presacral region with extensive bony destruction of the L5 vertebral body and sacrum (Figure 2). The patient was treated conservatively. However, the pain deteriorated 6 years later. MRI demonstrated enlargement of the tumor with erosion of the right L5 body to S1. A partial tumor excision was carried out using a posterior approach, and lumbopelvic fixation and bone grafting were also performed. The patient showed a complete recovery from the pain and no neurological deficit. The tumor did not show any enlargement during the follow-up period.

## Results

### Clinical presentations

**Figure 3.**
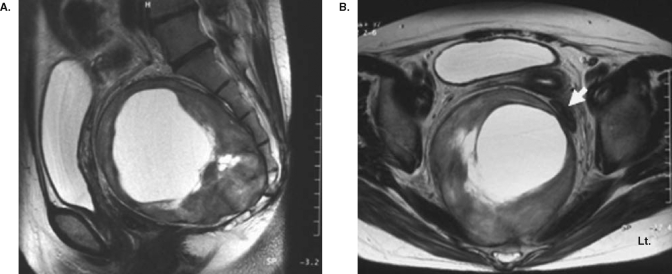
Sagittal image (A) and axial image (B) of T2-weighted MRI showing a giant tumor with a cystic component at the presacral retroperitoneal space displacing intrapelvic organs including the bladder and rectum (white arrow). To excise the tumor, the resection and re-anastomosis of the sigmoid colon was performed by the anterior approach.

A summary of the clinical figures and surgical details is shown in Table I. All patients were experiencing pain at the time of presentation. The duration of symptoms between onset and presentation ranged from 9 months to 23 years (average 11.2 years), except for one patient who presented with pain and sensory disturbances for 7 days. Pain was presented in the unilateral buttock area in three patients and extended to the lower extremity. The other three patients complained of unilateral leg pain. A neurological deficit was presented in five patients including minor motor weakness (grade 5/6 by manual muscle testing), sensory disturbances, and a decrease of the ankle tendon reflex. One patient (case 6) who presented with a retroperitoneal tumor did not show any neurological deficits, but complained of pollakiuria and constipation, because the bladder and rectum had been severely compressed by the large mass (Figure 3). Four patients had preoperative tissue diagnosis by needle biopsy. The mean observation time before surgery in the out-patient service was 3 years, ranging from 2 months to 7 years.

**Table I.** Clinical summary of the giant sacral and presacral schwannomas.

Case	Age/sex	Symptoms	Duration^a^	Intervention	Finding	Surgery	Complication	Outcome (follow-up in yrs)
1	57 F	Left leg pain	2 mth		Intraosseous type, size 5.5 cm	Posterior, enucleation	None	NER (12 years)
2	38 M	Right gluteal pain, right leg and foot numbness	23 yr	A	Dumb-bell type, size 15.0 cm	Combined^b^, subtotal	Erectile dysfunction, motor weakness	NER (11 years)
3	51 F	Right leg pain, right calf numbness	12 yr	AE	Dumb-bell type, size 12.0 cm	Combined, partial	Causalgia of right leg	Recurrence after 7 yrs, second surgery, NER (15 years)
4	43 F	Right leg pain, right leg numbness	3 yr	AE	Dumb-bell type, size 7.0 cm	Combined, subtotal	None	NER (3)
5	58 M	Buttock pain, right calf numbness	11 yr		Dumb-bell type, size 7.0 cm	Posterior, partial reconstruction	None	No evidence of growth (1.5 years)
6	49 F	Right buttock and leg pain, pollakiuria	23 yr	AE	Retroperitoneal type, size 11.4 cm	Anterior, subtotal	None	NER (0.5 year)

^a^Duration from onset of symptoms until surgery. ^b^Both anterior and posterior approach. A = angiogram; AE = angiogram with embolization; NER = no evidence of recurrence.

### Radiologic examination

A plain X-ray of the sacrum revealed osteolytic lesions with a sclerotic border in five patients. No bony change was observed in one patient (case 6). CT showed the details of bony involvement and presacral extension. Sacral osteolytic lesions were obvious in five patients. Two patients showed bony involvement of the L5 vertebral body (cases 2 and 5), and one patient showed sacroiliac joint involvement (case 5; Figure 2).

MRI revealed heterogeneous iso/high intensity on T1/T2 WI in all tumors. All tumors demonstrated a cystic lesion which showed obviously high intensity on T2 WI. Gd-DTPA enhancement was observed in all tumors. The extent of the tumor was demonstrated by MRI. A presacral tumor was demonstrated in five patients with a mean diameter of 9.6 cm, range 5.5–15.0 cm. Four tumors involving the spinal canal were located in the epidural space. Three tumors extended through the sacral body into the presacral region, and one tumor eroded the sacral body and formed a large intraosseous mass (case 1). One tumor was in the S1 foramen, expanded through the sacral body, and formed a presacral mass without epidural extension (case 4). One huge presacral tumor did not present with any bony extension (case 6; Figure 3). The tumors were classified as intraosseous type in one case (16%), dumb-bell type in four cases (66%), and retroperitoneal type in one case (16%).

### Surgery

The tumors were removed with a piecemeal subtotal excision leaving a part of the capsule for preservation of the nerves in three patients, a partial excision in two patients, and enucleation with the capsule remaining in one patient. Tumor removal was performed by the combination of an anterior and posterior approach in three patients. The tumor was removed by a posterior approach in two patients. An anterior approach was used in one patient, who presented with a huge presacral mass without bony involvement (case 6). In this case, the resection and re-anastomosis of the sigmoid colon was performed to excise the tumor (Figure 3). Lumbopelvic fixation was performed in one patient with extensive bony destruction (case 5; Figure 2). Preoperative embolization was performed in three patients. The mean surgical time was 7.8 hrs (2.9–10.6 hrs), and the mean intraoperative blood loss was 2572 g (range 483–5301 g). The pathological diagnosis was schwannoma in all cases, and malignant findings were not observed.

### Outcomes

There were no systemic complications. Pain decreased in all cases, and urinary disturbances also improved. One patient showed causalgia that was successfully treated by a caudal block (case 3). Although another patient experienced erectile dysfunction and motor weakness after surgery, both symptoms recovered after 2 years (case 2).

**Figure 4.**
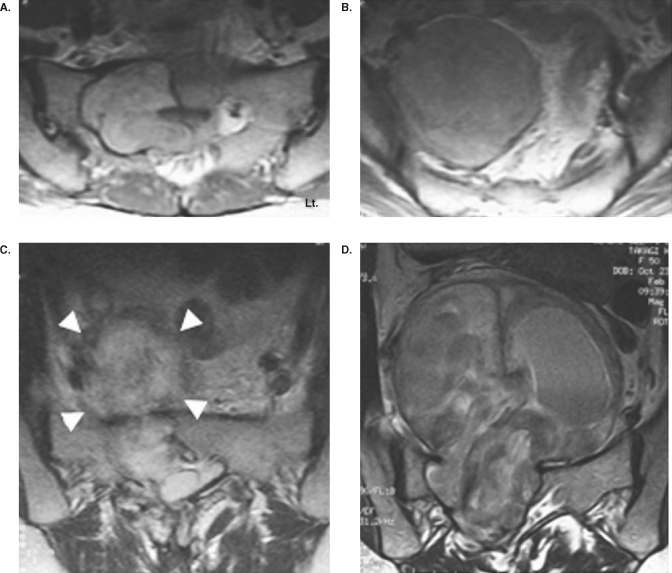
Axial image of T2-weighted MRI showing a big tumor extending from the spinal canal through the sacral body of S1 (A) to the presacral region (S2 level (B)) before surgery. The tumor was partially removed via a combination of the anterior and posterior approach. Axial image of T2-weighted MRI immediately after the surgery (C) showing a postoperative hematoma in the remaining tumor capsule (white arrow-head). Seven years after the surgery, the remaining tumor grew (D), and complete removal of the tumor with S1 root sacrifice was performed.

The tumor recurred in one patient (case 3; Figure 4). A 51-year-old female experienced severe pain in the right leg. The tumor was partially removed via a combination of an anterior and posterior approach. Seven years after the surgery, however, the remaining tumor grew, and the pain appeared again. A complete removal of the tumor with S1 root sacrifice was performed, and the pain was resolved.

No patient, including the patient who underwent the second surgery, presented with pain and obvious neurological deficit at the final follow-up.

## Discussion

A sacral schwannoma is uncommon and usually asymptomatic when it is small. The tumor is often found after presenting with pain and neurological symptoms (2,5,13–16). The current cases showed that three patients who presented with buttock pain then gradually presented with radiculopathy of one or more sacral roots. The other three patients complained of radicular pain to the legs from the beginning. The tumors have often reached an enormous size when they are discovered, and therefore they are usually difficult to manage.

MRI is helpful in detecting the specific characteristics of the tumor. It provides information about the size, exact location, and invasion of and relationship with other organs. Typically, a schwannoma is well encapsulated and solid when it is small, but may present cystic and necrotic changes in it when it is large. The purported mechanism of this phenomenon is that increasing tumor size results in vascular insufficiency. Hughes et al. (4) described the MRI characteristics of a presacral schwannoma as a homogeneous or heterogeneous/cystic appearance with well defined margin and iso/iso intensity or iso/high intensity on T1/T2 WI. A cystic appearance was demonstrated as an area of high intensity on T2 WI in all of the tumors in the current series.

The surgical approach is dependent upon the degree of intrasacral and retroperitoneal extension. The posterior approach should be used in cases of the tumor involving the spinal canal and intrasacrum. A tumor with a modest presacral component might be removed by the posterior approach. Generally, an anterior transabdominal or retroperitoneal approach should be performed for a presacral tumor to gain control of the vascular plexus that encompasses the tumor and to ensure identification and protection of the intrapelvic organs (14–16,18,19). Reconstruction of bony structures should be considered and preoperatively planned depending on the degree of sacral destruction and sacroiliac joint involvement. CT can be used to accurately detect the bony destruction caused by the tumor and should be part of the preoperative evaluation in all patients.

The optimal technique for the removal of a presacral and sacral schwannoma remains to be elucidated. Local recurrence and malignant transformation are extremely rare, so a piecemeal subtotal excision or simple enucleation should be the treatment of choice. At the same time, an aggressive approach with the aim of achieving a complete resection has also been reported (5,15). Piecemeal tumor excision was performed in all of the current cases. Recurrence was detected in only one patient (16%) who underwent partial excision in the first surgery. The tumor was removed completely in a second surgery, and the patient remained disease-free during the follow-up period. The need for an aggressive excision with the sacrifice of nerves should be carefully considered based upon the status of the patients, neurological findings, and tumor characteristics.

## Conclusion

Despite the limitations due to the small number of patients in the current cases, the results indicate as follows: 1) The duration of symptoms in a patient with a giant sacral schwannoma is very long, and pain is a dominant presenting symptom in all cases. 2) MRI is a useful diagnostic tool, and these tumors tend to show specific features. In the same way, CT clearly demonstrates bony destruction and should be used in preoperative planning. 3) A piecemeal subtotal tumor excision via the anterior, posterior, or combined approach can achieve a good outcome, avoiding unnecessary neurological deficit.

## References

[1] Whittaker LD, Pemberton JD (1938). Tumor ventral to the sacrum. Ann Surg.

[2] Klimo P, Rao G, Schmidt RH, Schmidt MH (2003). Nerve sheath tumors involving the sacrum: Case report and classification scheme. Neurosurg Focus.

[3] Turner ML, Mulhern CB, Dalinka MK (1981). Lesions of the sacrum. Differential diagnosis and radiological evaluation. JAMA.

[4] Hughes MJ, Thomas JM, Fisher C, Moskovic EC (2005). Imaging features of retroperitoneal and pelvic schwannomas. Clin Radiol.

[5] Theodosopoulos T, Stafyla VK, Tsiantoula P, Yiallourou A, Marinis A, Kondi-Pafitis A (2008). Special problems encountering surgical management of large retroperitoneal schwannomas. World J Surg Onc.

[6] Gosh BC, Gosh L, Huvos AG, Fortner JG (1973). Malignant schwannoma: A clinicopathological study. Cancer.

[7] Woodruff JM, Susin M, Godwin TA, Martini N, Erlandson RA (1981). Cellular schwannoma. A variety of schwannoma sometimes mistaken for a malignant tumor. Am J Surg Pathol.

[8] Regan JF, Juler GL, Schmutzer KJ (1977). Retroperitoneal neurilemoma. Am J Surg.

[9] Donnal JF, Backer ME, Mahony MS, Leight GS (1988). Benign retroperitoneal schwannoma. Urology.

[10] Schindler OS, Dixon JH (2002). Retroperitoneal giant schwannomas: Report on two cases and review of the literature. J Orthop Surg.

[11] Dede M, Yagci G, Yenen MC, Gorgulu S, Deveci MS, Cetiner S (2003). Retroperitoneal benign schwannoma: report of three cases and analysis of clinico-radiologic findings. Tohoku J Exp Med.

[12] Li Q, Gao C, Juzi JT, Hao X (2007). Analysis of 82 cases of retroperitoneal schwannoma. ANZ J Surg.

[13] Turkn PS, Peters N, Libbey WHJ (1992). Diagnosis and management of giant intrasacral schwannoma. Cancer.

[14] Salvant JB, Young HF (1994). Giant intrasacral schwannoma: an unusual cause of lumbrosacral radiculopathy. Surg Neurol.

[15] Abernathy CD, Onofrio BM, Scheithauer B, Pairolero PC, Shievs TC (1986). Surgical management of giant sacral schwannomas. J Neurosurg.

[16] Domíguez J, Lobato RD, Ramos A, Rivas JJ, Gómez PA, Castro S (1997). Giant intrasacral schwannomas: report of six cases. Acta Neurochir (Wien).

[17] Ozawa H, Kokubun S, Aizawa T, Hoshikawa T, Kawahara C (2007). Spinal dumbbell tumors: an analysis of a series of 118 cases. J Neurosurg Spine.

[18] Stewart RJ, Humphreys WG, Parks TG (1986). The presentation and management of presacral tumors. Br J Surg.

[19] Feldenzer JA, McGauley JL, McGillicuddy JE (1989). Sacral and presacral tumors: Problem in diagnosis and management. Neurosurgery.

